# Sjogren's syndrome: a neurological perspective

**DOI:** 10.1055/s-0043-1777105

**Published:** 2023-12-29

**Authors:** Orlando Grazianni Povoas Barsottini, Marianna Pinheiro Moraes de Moraes, Pedro Henrique Almeida Fraiman, Victor Hugo Rocha Marussi, Alexandre Wagner Silva de Souza, Pedro Braga Neto, Mariana Spitz

**Affiliations:** 1 Universidade Federal de São Paulo, Escola Paulista de Medicina, Departamento de Neurologia e Neurocirurgia, Setor de Neurologia Geral e Ataxias, São Paulo SP, Brazil.; 2 Hospital Beneficência Portuguesa, Setor de Neurorradiologia, São Paulo SP, Brazil.; 3 Universidade Federal de São Paulo, Escola Paulista de Medicina, Departamento de Medicina, Divisão de Reumatologia, São Paulo SP, Brazil.; 4 Sociedade Brasileira de Reumatologia, Comissão de Vasculites, São Paulo SP, Brazil.; 5 Universidade Federal do Ceará, Departamento de Medicina Clínica, Divisão de Neurologia, Fortaleza CE, Brazil.; 6 Universidade do Estado do Ceará, Centro de Ciências da Saúde, Fortaleza CE, Brazil.; 7 Universidade do Estado do Rio de Janeiro, Serviço de Neurologia, Rio de Janeiro RJ, Brazil.

**Keywords:** Sjogren's Syndrome, Autoimmune Diseases of the Nervous System, Síndrome de Sjogren, Doenças Autoimunes do Sistema Nervoso

## Abstract

Sjogren's syndrome (SS) is a complex autoimmune disease characterized by lymphocytic infiltration of salivary and lacrimal glands, resulting in sicca symptoms. Additionally, SS presents with neurological manifestations that significantly impact the nervous system. This review aims to provide a comprehensive overview of the neurological aspects of SSj, covering both the peripheral and central nervous system involvement, while emphasizing diagnosis, treatment, and prognosis.

## Resumo

A síndrome de Sjogren (SS) é uma doença autoimune complexa caracterizada pela infiltração linfocítica das glândulas salivares e lacrimais, resultando em sintomas sicca. Além disso, a SS apresenta manifestações neurológicas que afetam significativamente o sistema nervoso. Esta revisão tem como objetivo fornecer uma visão abrangente dos aspectos neurológicos da SSj, abordando tanto o envolvimento do sistema nervoso periférico quanto do central, com ênfase no diagnóstico, tratamento e prognóstico.

## INTRODUCTION

Sjögren's syndrome (SS) is a very complex and heterogenous rheumatic disease. It is characterized by an autoimmune lymphocytic infiltration of the salivary and lacrimal glands, causing sicca symptoms of mucosal structures. 1 However, the disease is often accompanied by extra-glandular manifestations. A wide spectrum of clinical manifestations with multi-systemic involvement, including the nervous system, has been described. 2 Notably, SS could affect both the peripheral and central nervous system (CNS) with variable prevalence and clinical manifestations. 3 Furthermore, patients with SS and neurologic involvement may present with different clinical features and prognoses when compared with patients with SS without neurologic involvement. 2 4

This review aims to describe and highlight the main neurological manifestations of SS, its treatment, and prognosis.

## METHODS

References included in this narrative review were obtained from PubMed searches conducted between May 2023 and July 2023. We searched for the following MeSH terms: “Sjögren's syndrome”' “Sjögren's disease,” “neurologic manifestations,” “nervous system,” “'neurological involvement” irrespective of publication date. Only articles that were published in English were reviewed. A total of 1,308 articles were found. Then, 39 articles were selected based on originality and relevance to the broad scope of this review. The authors recognize that the term “Sjögren's disease” may be the most appropriate for the condition, 5 however, due to its more common use, we decided to use the nomenclature “Sjögren's syndrome” in the text.

## DIAGNOSIS

SS is a chronic autoimmune disorder characterized by inflammatory T cell infiltration of exocrine glands, primarily lacrimal and salivary glands, resulting in the prototypical sicca syndrome, which refers to dryness of the eyes and mouth. 6 Additionally, SS may also affect other organs and systems, especially in its primary form, leading to a wide range of clinical features that may be classified into exocrine glandular and extra glandular manifestations. The incidence of SS is highest among women in their 50s-60s, but it may also affect adolescents, young adults, and men. 6 7

SS can manifest as a primary condition (i.e., primary SS) not associated with other diseases or as secondary SS, associated with other systemic autoimmune rheumatic diseases, particularly rheumatoid arthritis (RA) and systemic lupus erythematosus (SLE). The disease presentation includes a wide array of manifestations besides the sicca symptoms, and one of the key features for SS diagnosis is the presence of focal lymphocytic sialadenitis with a focus score ≥1–a cluster of at least 50 lymphocytes within a 4 mm ^2^ area of glandular tissue on labial salivary gland biopsy. Interestingly, a small group of patients may exhibit extra glandular manifestations and positive anti-Ro/SSA antibodies without experiencing xerostomia or xerophthalmia. 8

**Figure 1:**
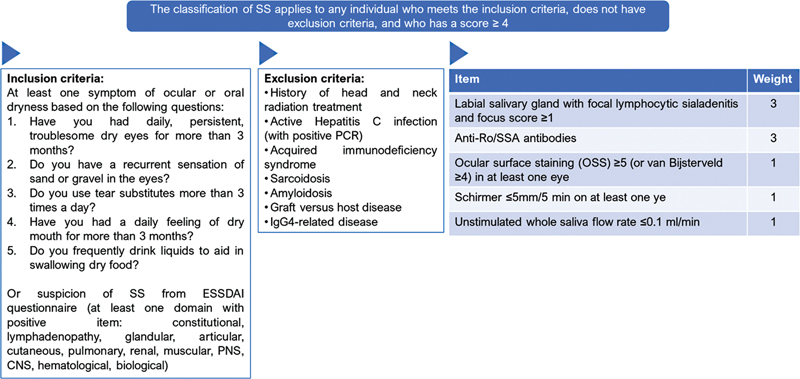
Step-by-Step Diagnosis of Sjogren's syndrome. Adapted from Shiboski et al. 9 Abbreviations: CNS, central nervous system; ESSDAI, EULAR Sjogren's syndrome disease activity index; PNS, peripheral nervous system.

The diagnosis of SS should be suspected in patients presenting with persistent symptoms of dry eyes and/or mouth, parotid gland enlargement, or abnormal results of certain serologic tests. These tests include the presence of anti-Ro/SSA antibodies with or without anti-La/SSB antibodies, rheumatoid factor, antinuclear antibodies, and hyperglobulinemia. The 2016 ACR-EULAR Classification Criteria, proposed by Shiboski et al., 9 were developed to include homogeneous sets of patients in clinical studies. These criteria should not be used for diagnostic purposes in clinical practice. In patients with suspected SS, after excluding mimics of SS, the diagnosis relies on the confirmation of objective findings of ocular and mouth dryness, salivary gland parenchymal changes, and confirming the autoimmune nature of the sicca syndrome. 9 ( Figure 1 )

Magnetic resonance imaging (MRI) or ultrasound (US) can be used to detect significant glandular parenchymal abnormalities characteristic of SS, which can aid in diagnosis. The detection of antinuclear antibodies (ANA) is the screening test to detect autoantibodies in a patient with suspected SS, and the fine-speckled pattern is associated with anti-SS-A/Ro and anti-SS-B/La antibodies. In some cases, patients may have anticentromere antibodies (in the absence of systemic sclerosis) or a positive rheumatoid factor. Although not included in the classification criteria, these laboratory findings can be considered as surrogate markers of SS in the appropriate clinical context. It is important to exercise caution when interpreting weakly positive antibody tests, especially if anti-La/SSB is the only positive result. In such cases, obtaining a labial salivary gland biopsy is appropriate to confirm the diagnosis of SS. It is also worth noting that a subset of individuals with systemic manifestations of SS may not exhibit sicca symptoms but can still be diagnosed based on a positive labial salivary gland biopsy and the presence of anti-Ro/SSA antibodies. 10

A thorough physical examination can provide important clinical clues for diagnosing SS. These include salivary gland enlargement, signs of salivary hypofunction (such as tooth decay), and a hyperlobulated tongue without filiform papillae. It is uncommon to observe lacrimal gland enlargement in SS, so the presence of this symptom should prompt consideration of alternative diagnoses such as IgG4-related disease, sarcoidosis, or lymphoma. Other suggestive extraglandular signs of SS include palpable purpura of the lower legs and peripheral neuropathy, especially in those presenting cryoglobulinemia. 6 8

In summary, we propose a step-based approach for patients with clinical suspicion of SS considering the various diagnostic tests and clinical features associated with the disease. This approach involves a combination of confirming the sicca syndrome, patient history, serologic tests, imaging techniques (MRI or US), and physical examination findings to accurately diagnose SS.

## PERIPHERAL NERVOUS SYSTEM MANIFESTATIONS

Peripheral neuropathies often accompany SS. However, in various series of patients with SS-associated neuropathy, it has been observed that over 90% of patients developed neuropathy before being diagnosed with the disease. 11 Additionally, more than a third of patients develop neuropathy prior to the onset of sicca symptoms, which are the well-recognized landmarks of the disease. 12 This poses a diagnostic challenge, but recognizing the common neuropathy patterns associated with SS is essential for determining a better diagnostic evaluation.

The well-recognized patterns of peripheral nervous system manifestations in SS are sensory ganglionopathy, painful small fiber neuropathy, trigeminal neuropathy, multiple mononeuropathies, multiple cranial neuropathies, polyradiculoneuropathies, and autonomic neuropathies. 11 13 14 15 16 17 The prevalence of these manifestations varies among case series, reflecting differences in the diagnostic criteria of SS and neuropathy. However, it is estimated that 5% of all SS patients have ganglionopathy, and 5–10% have small fiber neuropathy. 18 SS should be considered as a potential differential diagnosis in patients with sensory and sensorimotor symptoms, particularly in cases of sensory-only involvement like sensory ganglionopathy and small fiber neuropathy.

**Figure 2:**
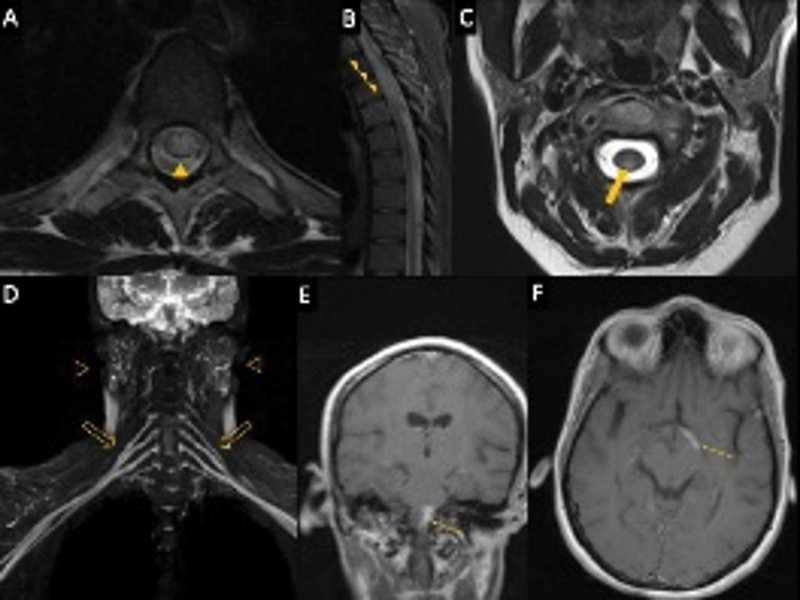
Neuroimaging findings of SS. (A, B) Axial and Sagittal T2WI showing extensive central spinal cord lesion (arrowheads) in a patient with NMOSD secondary to SS. (C), Axial T2WI revealing focal posterior lesion (thick arrow) in cervical spinal cord. (D) Coronal 3D STIR SPACE MIP (maximum intensity projection) displaying diffuse thickening of brachial plexus (hollow arrows) as well as diffuse enlargement within microcysts in parotid glands, a finding typical of SSj (hollow arrowheads). (E, F) showing brainstem and left optic radiation (thin arrows) enhancing lesions in two different patients.

Ganglionopathy is typically associated with SS, and is characterized by sensory ataxia, reduced or absent joint position, reduced or absent reflexes with normal strength, and selective damage to dorsal root ganglia ( Figure 2, C-D ). Nerve conduction studies show reduced or absent sensory nerve action potential. Patients are usually seronegative, and autoantibody serologies have low sensitivity. 18 19 Biopsy of the dorsal root ganglia reveals T lymphocyte infiltration and reduction of large fibers. 18 20 In salivary glands, intensive lymphocytic infiltration is observed (in a case series, over 90% of patients with sensory ganglionopathy had positive lip salivary gland biopsies). 19 This finding can be helpful when autoantibody serologies are negative but the etiology of ganglionopathy is unclear.

Small fiber neuropathy occurs in ∼20% of patients in the largest published series of SS-associated neuropathy. 11 It presents with subacute or chronic onset painful small fiber neuropathy accompanied by allodynia and hyperalgesia. Nerve conduction studies are usually unhelpful as they are generally normal. 13 A potential diagnostic clue in this context is the non-length-dependent distribution of symptoms, with early involvement of proximal parts of the limbs, trunk, or face. This finding is consistent with skin biopsy results, which reveal a reduction in intra-epidermal nerve fiber density simultaneously in the thighs (proximally) and in the feet (distally). 15 Similar to ganglionopathy, autoantibodies are usually negative and a lip salivary gland biopsy could yield an overall positivity of 81% in patients with suspected SS-related small neuropathy. 19

Autonomic dysfunction can manifest as orthostatic hypotension, heart arrhythmia, disorders of gastrointestinal motor activity, bladder dysfunction, secretomotor dysfunction, and pupillary abnormalities. It occurs in up to 50% of patients with SS. 11 The mechanism underlying autonomic dysfunction is unclear but seems connected to type 3 muscarinic receptor blocking. 13 A clinical clue to SS dysautonomia is the overlap with other peripheral nervous system manifestations of the syndrome. 18

Distal axonal sensory polyneuropathy is the most common form of neuropathy of SS. It has a chronic onset and slow progression. Commonly, it affects the lower limbs – the upper extremities involvement is uncommon (∼20%) – causing symmetric paresthesia and sensory disorders. It may be accompanied by small fiber neuropathy. 13 Its hallmark is biopsy displaying features of necrotic vascular inflammation of the nerve. 20 Multiple mononeuropathy prevalence varies between 12% to 50% in the case series. 11 21 22 23 As seen in polyneuropathy, vasculitis of the vessels nourishing the nerve trunks leading to nerve infarction is the hallmark of the process in SS. 20 Clinically, sensory and motor deficits could be observed in the distribution of the affected nerves with acute or subacute onset with notorious painful associated symptoms.

Among cranial nerve neuropathies, sensory trigeminal nerve involvement is the most commonly described type of cranial neuropathy in SS. Multiple cranial nerve involvement is rare but has already been described for the 3 ^rd^ , 5 ^th^ , 6 ^th^ , 7 ^th^ , 9 ^th^ , 10 ^th^ , and 12 ^th^ nerves in multiple combinations. Other isolated cranial nerve involvements described are 7 ^th^ and 8 ^th^ , presenting with hearing loss and vestibular symptoms. 11 24

## CENTRAL NERVOUS SYSTEM MANIFESTATIONS

SS is primarily associated with peripheral nervous system manifestations. However, CNS involvement has been reported in recent years. Virtually all structures may be affected, including the spinal cord, optic nerves, brainstem, cerebral hemispheres, and cerebellum ( Figure 2 A-F ). The most common clinical signs include aseptic meningitis, seizures, headache, cognitive decline, transverse myelitis, optic neuritis, ataxia, encephalopathy, and multiple sclerosis-like lesions. 13

Visuospatial and executive function impairment, attention, and memory deficits are the major cognitive manifestations of the disease. Brain MRI is unremarkable in most cases. Nevertheless, temporal and frontal hypoperfusion on SPECT has been described in some studies. 13 25

Aseptic meningitis is relatively common in SS patients and may be asymptomatic. Headache, meningeal involvement, seizures, and cranial nerve palsies are the most frequent signs in symptomatic cases. Lymphocytic pleocytosis with normal or slightly elevated protein levels is often found in the cerebrospinal fluid (CSF). 20

Approximately 10 to 20% of patients with SS may have multiple sclerosis-like lesions in the brain and spinal cord ( Figure 2, E-F ). Clinical manifestations in these cases include limb paresis, speech disorders, internuclear ophthalmoplegia, ataxia, and other signs resembling multiple sclerosis. Oligoclonal bands and increased IgG index may be seen in the CSF. Optic neuritis and transverse myelitis are also common. 13 20

Transverse myelitis is the most common medullary condition in patients with SS. Affected patients tend to display signs typical of myelopathic disorders, such as paraparesis and tetraparesis, bladder dysfunction, and sensory changes. Spinal MRI findings range from T2 hyperintense lesions, especially in the cervical region, to longitudinally extensive lesions similar to those seen in patients with aquaporin-4–positive Neuromyelitis Optica (NMO) ( Figure 2, A-B ). Other concurrent autoimmune diseases must be ruled out in patients with longitudinally extensive myelitis. 13 17 20

Bilateral retrobulbar optic neuritis has been extensively reported in SS patients and may be the first manifestation of the disease. Underlying pathogenic mechanisms appear to involve a combination of vasculitic and demyelinating inflammatory processes. 13 20 The association of NMO and MOGAD (myelin oligodendrocyte glycoprotein antibody disease) must be investigated in cases of SS-related optic neuritis. 26

In a study with 14 SS patients, Jacques et al 27 observed that most ataxia cases were of sensory type. However, cerebellar atrophy and cerebellar ataxia may also be observed in SS, mimicking a neurodegenerative disease.

## CONCOMITANT AUTOIMMUNE DISORDERS

SS has been described in association with a large variety of both organ-specific and systemic autoimmune diseases. 28 It is the most common connective tissue disease that appears in association with other autoimmune disorders, which may develop before or after SS diagnosis. 29

Autoimmune disorders that have been associated with SS are hypothyroidism, Graves' disease, celiac disease, autoimmune hepatitis, and primary biliary cholangitis. 30 SS is also frequently associated with other rheumatic diseases, in ∼30% of the cases, such as RA, SLE, scleroderma, and dermatopolymyositis. 31 In the original description of the disease by Henrik Sjögren in 1933, 13 (68.4%) out of 19 patients also had RA. 28 Rodriguez et al. 31 described that among 681 SS patients prospectively followed for a mean of 4.7 years, 30 developed a second autoimmune rheumatic disease, mostly RA, followed by scleroderma and SLE.

SS seems to influence the character and phenotype of the accompanying disease, either improving (less central nervous system symptoms in SLE) or exacerbating its course (lymphoma in RA). 29

SS is considered an important differential diagnosis of autoimmune demyelinating disorders, such as multiple sclerosis, NMOSD, and MOGAD. 32 Most NMOSD patients with comorbid SS are AQP4 positive. 33 Akaishi et al. 34 identified that comorbidity of SS and AQP4 NMOSD was ∼10–20% at the diagnosis of NMOSD and that more than 80% of patients with SS and acute CNS involvement were positive for serum AQP4-IgG. 34 Among NMOSD patients, 16% have anti-SSA and/or anti-SSB antibodies. 35 36 It has been demonstrated that patients with AQP4-IgG positive have higher titers of anti-SSA/SSB antibodies, a more acute course of SS, and a higher prevalence of other associated autoimmune disorders. 33 Transverse myelitis, which is a known presentation of CNS demyelinating disorders, occurs in 1 to 5% of SS patients. Patients with SS and transverse myelitis should be tested for AQP4-IgG and MOG antibodies. 26 35

## TREATMENT OF SJOGREN'S SYNDROME

SS treatment primarily focuses on the symptomatic relief of sicca symptoms and addressing systemic disease through immunosuppression. SS is often regarded as a true orphan disease in terms of therapeutic options due to the lack of consistently effective agents, despite advances in both basic and clinical research. 37 The complexity and heterogeneity of the disease further contribute to the difficulty in finding a universal treatment approach.

In addition to pharmacological interventions, non-pharmacological and preventive measures are essential components of SS management. These interventions aim to alleviate symptoms and improve patients' overall quality of life.

**Figure 3:**
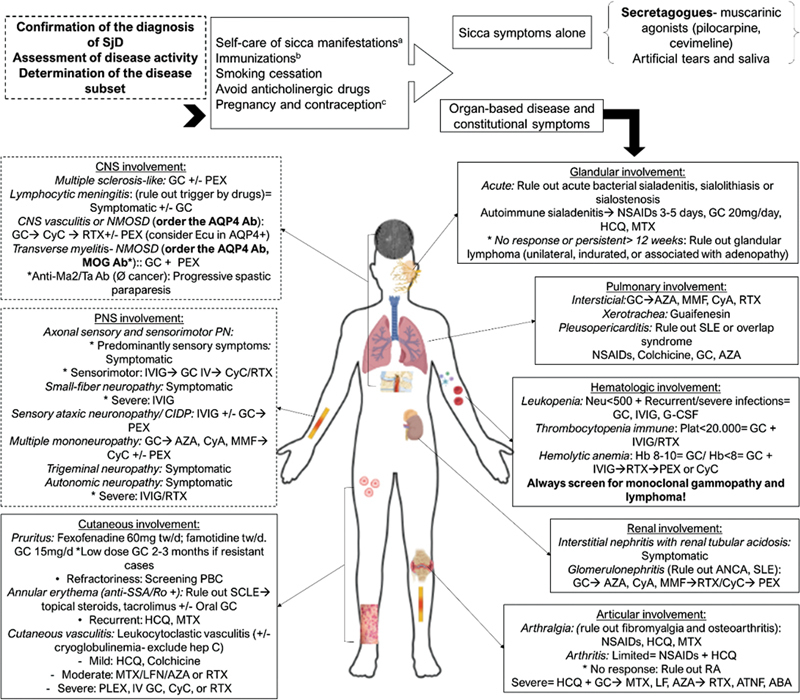
SS treatment. (a). Oral hygiene, knowledge about SjS and what symptoms require medical attention; (b). Appropriate immunization prior to immunosuppression; (c). Hormonal contraception is contraindicated in the case of antiphospholipid antibodies or a history of thrombosis. Abbreviations: AQP4 Ab, anti-aquaporin 4 antibody; CyA, ciclosporin A; CyC, cyclophosphamide [pulses 0.5 g/15 day (maximum six pulses)]; Ecu, eculizumab (doses 1–4: 900mg IV qWeek for first 4 weeks, followed by dose 5, 1200mg IV 1 week later, then 1200 mg IV q2Weeks); GC, glucocorticoids; HCQ, hydroxychloroquine (200mg/day); IVIg, intravenous immunoglobulins (0.4–2 g/kg 5 days); MOG, myelin oligodendrocyte glycoprotein; NMOSD, neuromyelitis optica spectrum disorder; NSAIDs, non-steroidal anti-inflammatory drugs (no longer than 7–10 days); PEX, plasma exchanges; RA, rheumatoid arthritis; RTX, rituximab [1 g/15 days (x2)]. Adapted from Ramos-Casals M, et al. 37

The choice of drug therapy for SS varies based on the specific organ manifestations and the severity of the disease, as determined by the EULAR Sjögren's Syndrome Disease Activity Index (ESSDAI). Treatment strategies may involve the use of immunosuppressive agents, such as corticosteroids, disease-modifying antirheumatic drugs (DMARDs), and biological agents, depending on the systemic involvement and individual patient characteristics. Figure 3 illustrates the main clinical manifestations and proposed treatments. 37 38 39

In conclusion, SS is an autoimmune systemic disease that may come into the neurologist's sight through a multitude of manifestations, affecting both peripheral and central nervous systems. The diagnosis is not always straightforward, so there must be a high index of suspicion, particularly in cases of sensory ganglionopathy, painful small fiber neuropathy, trigeminal neuropathy, multiple mononeuropathies, multiple cranial neuropathies, autonomic neuropathies, aseptic meningitis, transverse myelitis, and bilateral optic neuritis, in addition to demyelinating CNS lesions. The clinician should pursue symptoms of sicca syndrome, parotid gland enlargement, and serologic tests to confirm the diagnosis and establish the most adequate therapeutic management.
